# Proteomics Reveals the Obstruction of Cellular ATP Synthesis in the Ruminal Epithelium of Growth-Retarded Yaks

**DOI:** 10.3390/ani14081243

**Published:** 2024-04-22

**Authors:** Rui Hu, Ali Mujtaba Shah, Qiang Han, Jian Ma, Peng Dai, Yukun Meng, Quanhui Peng, Yahui Jiang, Xiangying Kong, Zhisheng Wang, Huawei Zou

**Affiliations:** 1 Low Carbon Breeding Cattle and Safety Production University Key Laboratory of Sichuan Province, Animal Nutrition Institute, Sichuan Agricultural University, Chengdu 611130, China; hurui14648@sicau.edu.cn (R.H.); 2018514001@stu.sicau.edu.cn (A.M.S.); 15982183167@163.com (Q.H.); crazyma0411@163.com (J.M.); dp114125@163.com (P.D.); pengquanhui@126.com (Q.P.); zswangsicau@126.com (Z.W.); 2 College of Animal Science and Technology, Sichuan Agricultural University, Chengdu 611130, China; 18004898129@163.com (Y.M.); jiangyahui.ff@163.com (Y.J.); 3 Haibei Demonstration Zone of Plateau Modern Ecological Animal Husbandry Science and Technology, Haibei 810299, China; 13909709088@163.com

**Keywords:** growth retardation, yaks, rumen, proteomic profile, energy production

## Abstract

**Simple Summary:**

The yak is the dominant livestock on the Tibetan plateau, providing milk, meat, fur, and income for the local residents; however growth-retarded yaks are of a high proportion and reduce the yak farming profitability. Our study found that the obstruction of energy metabolism and ATP synthesis in ruminal epithelium contributed to the growth retardation of yaks. It provided a fundamental understanding of how the energy metabolism of ruminal epithelium affected the growth retardation of yaks and provided new ideas for improving rumen health and the growth performance of growth-retarded yaks through nutritive regulation methods.

**Abstract:**

Growth-retarded yaks are of a high proportion on the Tibetan plateau and reduce the economic income of farmers. Our previous studies discovered a maldevelopment in the ruminal epithelium of growth-retarded yaks, but the molecular mechanisms are still unclear. This study aimed to reveal how the proteomic profile in the ruminal epithelium contributed to the growth retardation of yaks. The proteome of the ruminal epithelium was detected using a high-resolution mass spectrometer. There were 52 proteins significantly differently expressed between the ruminal epithelium of growth-retarded yaks and growth-normal yaks, with 32 downregulated and 20 upregulated in growth-retarded yaks. Functional analysis showed the differently expressed proteins involved in the synthesis and degradation of ketone bodies (*p* = 0.012), propanoate metabolism (*p* = 0.018), pyruvate metabolism (*p* = 0.020), and mineral absorption (*p* = 0.024). The protein expressions of SLC26A3 and FTH1, enriched in the mineral absorption, were significantly downregulated in growth-retarded yaks. The key enzymes ACAT2 and HMGCS2 enriched in ketone bodies synthesis and key enzyme PCCA enriched in propanoate metabolism had lower protein expressions in the ruminal epithelium of growth-retarded yaks. The ATP concentration and relative mitochondrial DNA copy number in the ruminal epithelium of growth-normal yaks were dramatically higher than those of growth-retarded yaks (*p* < 0.05). The activities of citrate synthase (CS), the α-ketoglutarate dehydrogenase complex (α-KGDHC), isocitrate dehydrogenase (ICD) in the tricarboxylic acid cycle (TCA), and the mitochondrial respiratory chain complex (MRCC) were significantly decreased in ruminal epithelium of growth-retarded yaks compared to growth-normal yaks (*p* < 0.05). The mRNA expressions of *COQ9*, *COX4*, and *LDHA*, which are the encoding genes in MRCC I, IV and anaerobic respiration, were also significantly decreased in the ruminal epithelium of growth-retarded yaks (*p* < 0.05). Correlation analysis revealed that the average daily gain (ADG) was significantly positively correlated to the relative mitochondrial DNA copy number (*p* < 0.01, r = 0.772) and ATP concentration (*p* < 0.01, r = 0.728) in the ruminal epithelium, respectively. The ruminal weight was positively correlated to the relative mitochondrial DNA copy number (*p* < 0.05, r = 0.631) and ATP concentration in ruminal epithelium (*p* < 0.01, r = 0.957), respectively. The ruminal papillae had a significant positive correlation with ATP concentration in ruminal epithelium (*p* < 0.01, r = 0.770). These results suggested that growth-retarded yaks had a lower VFA metabolism, ketone bodies synthesis, ion absorption, and ATP synthesis in the ruminal epithelium; it also indicated that the growth retardation of yaks is related to the obstruction of cellular ATP synthesis in rumen epithelial cells.

## 1. Introduction

The yak (*Bos grunniens*) is the dominant livestock on the Tibetan plateau above a 3000 m altitude. Yaks provide people with milk, meat, and fur, generating the local residents’ major economic income. Currently, yaks are mainly fed under the grazing production system, with little supplementing of concentrate feed. Because of the harsh environment, such as the long-term cold season and short growing season of the pasture, yaks suffer from severe shortages in foraging and malnutrition in the cold season year after year. Meanwhile, yaks are seasonal reproduction animals, mating in the warm season and delivering after about a 260-day pregnancy period. Therefore, most of the yaks’ pregnancy and juvenile periods are distributed in the long-term cold season. Malnutrition in the early life of yaks always inhibits their development and growth performance; therefore, growth-retarded yaks make up a high proportion of the Tibetan plateau, reducing the yak farming profitability.

Previous studies have found that growth retarded-cattle or yaks have lower secretions of somatotropic axis hormones [1], maldevelopment of the digestive tract [2], and a disrupted gastrointestinal bacterial community [3]. The growth performance and rumen morphology of growth-retarded yaks were studied in our previous study [2], which found that the average daily gain (ADG) of growth-normal yaks was almost three times higher than that of growth-retarded yaks. The papillae surface area and papillae height of ruminal epithelium in growth-retarded yaks were dramatically lower than those of growth-normal yaks. The expressions of tight junction proteins, such as CLDN1, OCLN, and ZO1, were dramatically down-regulated in the ruminal epithelium of growth-retarded yaks. The rumen plays important roles in feed digestion and nutrient absorption, which provides 70% of the metabolic energy requirements for ruminants through absorptions and the metabolism of volatile fatty acids (VFAs). Furthermore, the VFAs are also major energetic substrates for ATP synthesis in the ruminal epithelium [4], and ATP is an important energetic source for barrier function, cell proliferation, nutrient absorption, and immune function in the gastrointestinal epithelium. Cattle with higher feed efficiency had a higher rate of mitochondrial respiration in muscle [5], greater oxidative phosphorylation in liver mitochondria [6], and higher gene expressions in oxidative phosphorylation of the ruminal epithelium [7]. However, as the important digestive organs, there are few studies systematically investigating the proteomic profile and how ATP production in the ruminal epithelium contributes to the growth retardation of yaks.

Therefore, we hypothesized that growth-retarded yaks would have a lower ability of energy metabolism and production in the ruminal epithelium. Our study aimed at comparing the differences in proteomic profile and ATP generated between the ruminal epithelium of growth-retarded yaks and growth-normal yaks using proteomic sequencing, attempting to provide a novel insight into how ruminal functions contributed to the growth retardation of yaks.

## 2. Material and Methods

### 2.1. Animal Ethics Approval

The experimental procedures were conducted according to the Regulations for the Administration of Laboratory Animals (the State Council of China, 2017) and approved by the Institutional Animal Care and Use Committee of Sichuan Agricultural University (Approval No. 20200031).

### 2.2. Experimental Design

The detailed experimental design was described in the previous study [2]. Briefly, a growth-retarded yak was defined as having the yak’s body weight (BW) distributed in the lowest 10% of the yak population of the same age and breed [2]. Eight 16-month-old male Qinghai Plateau yaks (BW 74.0 ± 6.41 kg) were chosen as the growth-retarded yak group (GRY), while another eight male yaks (BW 112.0 ± 4.03 kg) of the same breed and age were chosen as the growth-normal yak group (GNY). All yaks were grazed on the same grassland at an altitude of 3200 m in the Qinghai Province of China. The dominant grass varieties and nutrient levels of grassland were described in the previous study [2]. The main natural grasses in the pasture are Elymus nutans, Kobresia humilis, Kobresia pygmaea, and Carex moorcroftii. The nutrient levels of the natural grass are presented as follows (dry basis): 5.02% crude protein, 53.1% neutral detergent fiber, 34.3% acid detergent fiber, 2.61% ether extract, and 11.2% crude ash. After a 2-month experimental period, the average daily gain (ADG) of yaks was calculated as 0.11 kg/d for GRY and 0.30 kg/d for GNY [2]. The ADG of GRY was 0.11 kg/d, whereas the ADG of GNY was 0.30 kg/d [2].

### 2.3. Sample Collection

Six yaks from each group were chosen randomly and slaughtered according to the standard procedure of China (GB/T 19477-2018) [8] after the feeding experiment. Approximately 2.0 cm × 2.0 cm of the ruminal epithelium was cut from the ventral sac of each rumen, put in a 1.5 mL freezing tube after being washed with 4 °C PBS, then directly frozen in liquid nitrogen.

### 2.4. Mass Spectrometry

The rumen epithelia tissues were analyzed by the high-resolution mass spectrometer. Firstly, total proteins were extracted from ruminal epithelium samples. After the concentrations of proteins were detected by a Bradford protein assay kit (Chemical Book, Shanghai, China), the proteins were enzymolysized into peptides. Briefly, each protein sample (100 μg) was diluted, and the enzyme solution, including Trypsion enzyme (μg): substrate protein (μg) = 1:20, was added and then vortexed, centrifuged at 1000× *g* for 1 min, and incubated at 37 °C for 5 h. The peptides were dissolved in the mobile phase A (0.1% formic acid and 2% ACN) and centrifuged at 19,000× *g* for 15 min. The supernatant was analyzed by the Thermo UltiMate 3000 UHPLC (Thermo Scientific, Rockford, IL, USA). The samples were firstly enriched in a trap column and desalted and entered the C18 column (25 cm column length, 3 μm column size, 75 μm internal diameter). The separated flow rate was 300 mL/min with the following effective gradient: 5% mobile phase B (0.1% formic acid and 98% ACN) at 0–5 min, mobile phase B linearly rose from 5% to 25% at 5–45 min, rose from 25% to 35% at 45~50 min, rose from 35% to 80% at 50~52 min, stabilized at 80% at 52~54 min, then decreased to 5% at 54~60 min. The mass spectrometer was directly linked to the end of the nanoliter liquid phase separator.

The separated peptides of liquid phase chromatography were passed to the high-resolution mass spectrometer (Triple TOF 5600 SCIEX, Framingham, MA, USA). The mass spectrometer’s parameters were followed: the ion source spray voltage was 2300 V, nitrogen pressure was 35 psi, and spray interface temperature was 150 °C. Scanning in high sensitivity mode, the parameters were set as 250 ms of scan cumulative time and 350~1500 Da of scan quality.

### 2.5. Bioinformatic Analysis

Raw data was analyzed using the MaxQuant 1.5.3.30 integrated Andromeda engine. Filtering was implemented with a PSM-level false discovery rate (FDR) ≤ 1% at the spectrum level and with the protein-level FDR ≤ 1% at the protein level. Then, the peak areas were extracted and protein quantitation was calculated using MaxQuant. The multiples of differences in the proteins of GRY and GNY were calculated, and Welch’s *t*-test was used to perform the significance test. The significantly differently expressed protein (DEPs) criteria were set as the *p*-value < 0.05 and multiple of difference > 1.5. Finally, the functional enrichment analysis was completed by Gene Ontology (GO) annotation and Kyoto Encyclopedia of Genes and Genomes (KEGG) annotation using the online tool, OmicsBean (http://www.omicsbean.cn/ accessed on 12 May 2023).

### 2.6. Relative Mitochondrial DNA Copy Number

The relative mitochondrial DNA copy number per cell was detected by the qPCR ratio of mitochondrial DNA (mtDNA) to nuclear DNA (nDNA). Briefly, the NADH dehydrogenase subunit 1 (ND1) gene was selected as the mitochondrial DNA target, and the DEAD box polypeptide 3 Y-linked (DDX3Y) gene was selected as the nuclear DNA target. The primer sequences of ND1 and DDX3Y were listed as follows: ND1-F: GAACCACTACGACCCGCTACA. ND1-R: GAGTTGGAAGCTCAGCCTGATC. DDX3Y-F: ATCGTGGGCGGAATGAGTGT. DDX3Y-R: CTTGGTGGAAGCGGTTTTGA [7]. Firstly, total DNA was extracted from each tissue sample using the bead beating method [7]. The DNA concentrations were detected by the Scandrop100 (AnalytikJenaAG, Jena, Germany). Secondly, PCR was performed to amplify the nDNA and mtDNA. The PCR mix consisted of 25 μL 2 × Tap Master Mix, 1 μL forward primer, 1 μL reverse primer, 1 μL template DNA, and 1 μL RNase free ddH_2_O. The PCR procedure was 1 cycle of 94 °C for 5 min, 30 cycles of 94 °C for 30 s, 62 °C for 30 s, 72 °C for 30 s, and 1 cycle of 72 °C for 7 min. The PCR products were purified by agarose gel electrophoresis using the HiPure Gel Pure DNA Micro Kits (Magen, Guangzhou, China), and the concentrations were detected by Scandrop100 (AnalytikJenaAG, Jena, Germany). The standard curve was created with 10-fold dilutions of each DNA. Finally, the relative mitochondrial DNA copy number was calculated according to the method described in the previous study [7].

### 2.7. Metabolite Concentrations in ATP Synthesis

The concentration of lactate in rumen epithelial tissues was determined by the lactic acid kit (BIOLAB, Beijing, China) according to the operating instructions. The concentrations of α-Ketoglutarate, succinate, ATP, ADP, and AMP were detected using a high-performance liquid chromatograph (HPLC) (Rigol L3000, Beijing, China). Additionally, 0.1 g of each tissue sample was used for HPLC analysis according to the methods of the previous study [9]. The column temperature of the reversed-phase C18 column was 30 °C, and the detection wavelength was 254 nm. Mobile phase A was made of 11.283 g Na_2_HPO_4_12H_2_O and 10.689 g NaH_2_PO_4_, and mobile phase B was methanol. The flow rate of the mobile phase was set as 0.8 mL/min. The standard substances of succinate, α-Ketoglutarate, ATP, ADP, and AMP were purchased from SIGMA (St. Louis, MO, USA).

### 2.8. The Activity of Key Enzymes of MRCC and LDHA

The activities of key enzymes included citrate synthase (CS), α-ketoglutarate dehydrogenase complex (α-KGDHC), and isocitrate dehydrogenase (ICD) in the TCA; mitochondrial respiratory chain complex (MRCC) I to V and lactate dehydrogenase A (LDHA) were detected using ELISA kits (BIOLAB, Beijing, China) according to the operating instruction.

### 2.9. Quantitative Real-Time (qPCR) Detected mRNA Expressions of MRCC and LDHA

Total RNA was extracted from the ruminal epithelium using the TRIzol reagent (Invitrogen, Carlsbad, CA, USA). An OD 260/280 value greater than 1.8 was required. RNA integrity was detected by an Agilent 2100 Bioanalyzer, and the RIN (RNA integrity number) value of the sequencing sample was needed to be greater than 7 to ensure that the quality of RNA extraction was suitable for subsequent experiments. In addition, the efficiency of qPCR detection for the target gene and the housekeeping gene used was indicated in Table 1. The RNA concentrations were detected using Scandrop100 (AnalytikJenaAG, Jena, Germany). Then, RNA was reverse transcribed to cDNA using an RT EasyTM II kit (Foregene, Chengdu, China). The primers of Coenzyme Q9 (COQ9), Cytochrome c oxidase subtype IV (COX4), ATP synthase F(0) complex subunit B1 (ATP5F1), LDHA and 18S were designed using Primer-Blast online (https://blast.ncbi.nlm.nih.gov/Blast.cgi accessed on 18 July 2023) (Table 1). The qPCR was analyzed in triplicate using the SYBR^®^ Green Realtime PCR kit (Takara Biotechnology, Dalian, China) according to the operating manual. Briefly, the reaction system consisted of 10 μL SYBR^®^ Green Realtime PCR Master Mix, 1 μL forward primer, 1 μL reverse primer, 4 μL template DNA, and 4 μL PCR grade water. The qPCR program was set as preincubation at 95 °C for 2 min, followed by 39 cycles of denaturation at 95 °C for 15 s and annealing/extension at 58 °C for 30 s. Finally, a melting curve analysis was performed. The expressions of GAPDH and 18S were detected in this study, and 18S was chosen as the housekeeping gene because of its more stable expression. Relative expressions of target genes were calculated by the 2^−ΔΔct^ method.

### 2.10. Statistical Analysis

An independent-samples *t*-test of SPSS (Version 19.0) was used to analyze the significant differences in the mitochondrial DNA copy number, metabolite concentrations, enzyme activities, and relative mRNA expressions. The data of ADG, rumen weight, and ruminal epithelium morphological parameters were shown in our previous study [2]. In this study, Pearson correlation of SPSS (Version 19.0) was used to analyze the correlation of ADG, ruminal epithelium morphological parameters, and ATP production. The results are presented as mean ± SEM, and *p* < 0.05 was regarded as the significant difference.

## 3. Results

### 3.1. Significantly Differentially Expressed Proteins

A total of 2048 proteins were identified in this study, and 52 significantly different expressed proteins (DEPs) were found in the ruminal epithelium between GRY and GNY. The results showed that 32 DEPs were downregulated and 20 DEPs were upregulated in the ruminal epithelium of GRY compared to GNY (Table 2). The most upregulated DEPs in the ruminal epithelium of GRY were collagen alpha-1(VII) chain (COL7A1), urea transporter 1 (SLC14A1), and alpha-endosulfan (ENSA), while the most downregulated DEPs in GRY were V-type proton ATPase subunit D (ATP6V1D), glutathione peroxidase 1 (GPX1), 2-oxo isovalerate dehydrogenase subunit beta, mitochondrial (BCKDHB), V-type proton ATPase catalytic subunit A (ATP6V1A) and hydroxymethylglutaryl-CoA synthase, as well as mitochondrial (HMGCS2).

### 3.2. Gene Ontology (GO) Analysis of DEPs

The GO analysis showed that two significantly enriched terms were annotated to cellular component (CC), four significantly enriched terms were annotated to molecular function (MF), and 27 significantly enriched terms were annotated to biological process (BP). In the cellular component, the significantly enriched terms were proton-transporting V-type ATPase complex (*p* = 0.024) and plasma membrane (*p* = 0.042). In the molecular function, the significantly enriched terms included steroid hormone receptor binding (*p* = 0.017), nuclear hormone receptor binding (*p* = 0.032), hormone receptor binding (*p* = 0.038), and active transmembrane transporter activity (*p* = 0.039). In the biological process, the representative terms were transition metal ion transport (*p* = 0.003), intracellular steroid hormone receptor signaling pathway (*p* = 0.017), cellular ion homeostasis (*p* = 0.028), and ion transport (*p* = 0.038) (Supplementary Table S1).

### 3.3. Kyoto Encyclopedia of Genes and Genomes (KEGG) Analysis of DEPs

The significantly enriched KEGG of the DEPs is shown in Table 3. In total, 14 KEGG pathways were significantly enriched (*p* < 0.05), of which 11 pathways belonged to the metabolism pathway, mainly including valine, leucine and isoleucine degradation (*p* = 0.002), thiamine metabolism (*p* = 0.006), synthesis and degradation of ketone bodies (*p* = 0.012), propanoate metabolism (*p* = 0.018), pyruvate metabolism (*p* = 0.020) and fatty acid degradation (*p* = 0.022). Another two KEGG pathways were involved in the organismal systems, including mineral absorption (*p* = 0.024) and collecting duct acid secretion (*p* = 0.034). The protein expression of HMGCS2 and ACAT2, enriched in the synthesis and degradation of ketone bodies pathway, was significantly decreased in the ruminal epithelium of GRY. Protein expressions of BCKDHB, ACAT2, PCCA, enriched in the propanoate metabolism pathway, were significantly reduced in GRY. The protein expressions of SLC26A3 and FTH1, enriched in mineral absorption, were significantly decreased in GRY.

### 3.4. Mitochondrial Number and Metabolite Concentrations of ATP Synthesis

The relative mitochondrial DNA copy number in the ruminal epithelium of GRY was significantly decreased compared to GNY (*p* < 0.01) (Table 4). The results showed that GRY had a higher α-ketoglutarate concentration in ruminal epithelium when compared to GNY (*p* < 0.05), whereas the succinate concentration showed no significant difference between GRY and GNY (*p* > 0.05). The concentrations of ATP, AMP, and lactate in the ruminal epithelium of GRY were significantly lower compared to GNY (*p* < 0.05).

### 3.5. Enzymatic Activity in a Tricarboxylic Acid Cycle and Electron Transport Chain

Enzymatic activities in ATP production are shown in Table 5. The enzymatic activities of CS, ICD, and α-KGDHC in a tricarboxylic acid cycle (TCA) were significantly lower in the ruminal epithelium of GRY than those of GNY (*p* < 0.05). The enzymatic activities of mitochondrial respiratory chain complex (MRCC) I to V in the ruminal epithelium of GRY were significantly decreased when compared to GNY (*p* < 0.05). The enzymatic activity of lactate dehydrogenase (LDHA) was also lower in the ruminal epithelium of GRY than that of GNY (*p* < 0.05).

### 3.6. mRNA Expressions of MRCC and LDHA

The relative mRNA expressions of *COQ9*, *COX4*, and *LDHA*, which are the representative encoding genes in MRCC I, IV, and anaerobic respiration, were significantly lower in the ruminal epithelium of GRY than those of GNY (*p* < 0.05) (Table 6).

### 3.7. Correlation Analysis between Ruminal Epithelial Morphology and ATP Synthesis

Correlation analysis is shown in Table 7. The ADG had a significant positive correlation with the relative mitochondrial DNA copy number (*p* < 0.01) and ATP concentration in ruminal epithelium (*p* < 0.01), respectively. The rumen weight had a significant positive correlation with the relative mitochondrial DNA copy number (*p* < 0.05) and had a highly significant positive correlation with the ATP concentration (*p* < 0.01) in ruminal epithelium. The height of ruminal papillae had a significant positive correlation with the ATP concentration in the ruminal epithelium (*p* < 0.01).

## 4. Discussion

### 4.1. Proteome Analysis

The ADG of GNY was almost three times higher than that of GRY. Because yaks in this study were grazed on the natural pasture, the feed intake of these animals is difficult to measure. Our previous study found that ADG and dry matter intake of GRY were significantly lower than those of GNY [10]. The nutrients digestibility of growth retarded cattle were also significantly lower than those of normal growth cattle [11]. These results indicated that the lower feed intake and nutritional digestibility potentially contributed to the growth retardation of ruminants. The rumen is the important digestive organ of ruminants. Our previous studies proved that GRY had significantly lower papillae height, barrier dysfunction, and inflammation in the ruminal epithelium [2], and this study used proteomic analysis to reveal the functional differences in the ruminal epithelium between GRY and GNY. According to KEGG analysis of the DEPs, 11 of the 14 significantly enriched pathways belonged to metabolism, suggesting that the functional difference between GRY and GNY mainly focused on the metabolism of ruminal epithelium.

The ruminal epithelium is the major site used to absorb nutrients, and it provides 70% of the energy requirement of ruminants through VFA absorption. In this study, results showed that chloride anion exchanger (SLC26A3), type A and D of the V-type proton ATPase had significantly lower expression in the ruminal epithelium of GRY. SLC26A3 belongs to the family of solute carrier proteins and is the ruminal epithelium’s dominant absorption protein. A previous study found that beef cattle with a higher feed conservation rate had significantly higher mRNA expressions of SLC26A3 compared to cattle with a low feed conservation rate [12]. V-type proton ATPase is a multi-subunit protein that transfers protons depending on energy produced by ATP hydrolysis, which plays an important role in nutrient absorption and balancing of the intracellular and extracellular environments. A previous study showed that growth-retarded lambs had lower VFA concentrations in the rumen when fed the same diets [13], indicating that lower protein expressions of nutrient transporters in the ruminal epithelium were also the cause of the lower VFA production of GRY.

After absorption, VFA metabolisms are essential for epithelial development and functioning of the rumen. The anabolism of the ketone body is the hallmark of a mature ruminal epithelium [4]. The 95% of absorbed butyrate is metabolized into ketone bodies to provide energy sources in the ruminal epithelium [14]. The HMGCS2 and ACAT2 are the rate-limiting enzymes of ketone body synthesis in the ruminal epithelium [15,16]. Previous studies found that gene expression of HMGCS2 was higher in the ruminal epithelium of post-weaning ruminants than in pre-weaning ruminants [17,18]. Expression of the ACAT2 gene was significantly upregulated in ruminal epithelium when the cattle diet transferred from high forage to high grain [19]. Our results found that protein expressions of HMGCS2 and ACAT2 downregulated in the ruminal epithelium of GRY, which was significantly enriched in the synthesis and degradation of ketone bodies, suggesting that GRY had a lower ketogenic capacity in the ruminal epithelium. The results also found that protein expressions of PCCA, enriched in the propanoate metabolism pathway, were significantly downregulated in the ruminal epithelium of GRY. PCCA is the key enzyme involved in propionate metabolism in mitochondria, which converts propionate to propionyl-CoA [20]. Propionyl-CoA converting to succinyl-coenzyme A was the important energy substance for the TCA cycle in ruminal epithelium cells. The PCCA mRNA expression significantly increased in the ruminal epithelium of dairy cattle supplemented concentrate rapidly, suggesting increased ATP synthesis in ruminal epithelium after supplemented concentrate [21]. Our results indicated that the ATP synthesis was downregulated in the rumen epithelium of GRY.

### 4.2. ATP Synthesis in Ruminal Epithelium

Consistently, our results showed that GRY had a lower relative mitochondrial DNA copy number and ATP synthesis in the ruminal epithelium. The number of mitochondria in cells has a positive correlation with ATP synthesis [22]. Previous studies also found that beef cattle with high feed efficiency had a higher mitochondrial copy number in the ruminal epithelium [7], higher oxidative phosphorylation (OXPHOS) in liver mitochondrial [6], and higher mitochondrial respiration in muscle [5]. The TCA cycle and mitochondrial respiratory chain are the most efficient ways of synthesizing ATP. Our results showed that the activities of key enzymes CS, ICD, and α-KGDHC in the TCA cycle and enzyme activities of MRCC were significantly lower in the ruminal epithelium of GRY, indicating the mitochondrial respiration inhibited in the ruminal epithelium of GRY. We also found a significantly higher concentration of α-ketoglutarate, the substrate of the α-KGDHC enzyme, in the ruminal epithelium of GRY. This result was consistent with the lower enzymatic activity of α-KGDHC. Lactate is the product of anaerobic respiration, and the LDH-A is the key enzyme to catalyze pyruvate to lactate. Our results found that the lactate concentration and enzyme activity of LDH-A in the ruminal epithelium of GRY were significantly lower than those of GNY, suggesting that anaerobic respiration also obstructed in GRY. Therefore, the results indicated the ATP synthesis is insufficient in the ruminal epithelium of GRY.

The *COQ9* and *COX4* are the subunits of MRCC I, IV, and V, respectively. *COQ9* is an important coenzyme Q that stabilizes the function of MRCC I and transports electrons. The content of *COX4* was positively related to mitochondrial density and ATP production [23,24]. Glutamine deprivation downregulated the *COQ9* and *COX4* protein expressions and inhibited the ATP production in HepG2 cell lines [25]. *ATP5F1* is the subunit of MRCC V, also named the ATP synthase, a multi-subunit enzyme on the mitochondrial membrane for synthesizing ATP [26]. This study found that mRNA expressions of *COQ9* and *COX4* were significantly downregulated in the ruminal epithelium of GRY, suggesting the inhibition of mitochondrial ATP synthesis. Meanwhile, the significantly lower expression of the *LDHA* gene also indicated lower ATP production from the cellular anaerobic respiration pathway.

Cellular energy synthesis is important for the ruminal epithelium’s morphological development, nutrient absorption, and barrier function. Cellular mitosis and proliferation are the basis for the development of the gastrointestinal tract, and are highly energy-consuming processes. Energy deprivation inhibited the G1-S cell cycle in multiple tissues of Drosophila [27]; therefore, combined with our previous findings, we found that rumen weight and nipple height were significantly positively correlated with cellular ATP production. The recent study also found that β-hydroxybutyrate, as the dominant metabolic ketone, facilitated rumen growth by promoting the oxidative phosphorylation to produce ATP [28], and it also suggested that the lower ketogenesis and ATP production contributed to the maldevelopment of the ruminal epithelium of growth-retarded yaks. A healthy rumen is fundamental to a high feed conversion ratio, and our results also showed a significant positive relationship between ADG and ATP production in yaks.

## 5. Conclusions

The proteome analysis showed the DEPs in the ruminal epithelium between the GRY and GNY mainly focused on cellular energy metabolism, including ketone body metabolism, propanoate metabolism, and pyruvate metabolism. The decreased expressions of HMGCS2 and ACAT2 indicated the obstruction of the VFA metabolism and ketone body synthesis in the ruminal epithelium of GRY. The reduced expressions of SLC26A3, ATP6V1D, and ATP6V1A indicated the inefficient VFA and ion absorption in the ruminal epithelium of GRY. The results also indicated both down-regulated mitochondrial respiratory and anaerobic respiration in the ruminal epithelium of GRY. The ADG, ruminal weight, and papillae height were positively correlated to ATP concentration in the ruminal epithelium of yaks. Therefore, our results suggested that obstruction of cellular ATP in the ruminal epithelium contributed to the growth retardation of yaks. This provides a fundamental understanding of the growth retardation of yaks and gives new ideas to improve the rumen health and growth performance of growth-retarded yaks.

## Figures and Tables

**Table 1:** Primer information of qPCR genes.

Gene Name	Accession Number	Forward and Reverse Primer Sequence (5′-3′)	Product Length (bp)
*18S*	XM_005891364.2	F: TCCTGCTCGGACCAC	147
		R: CAATGCCAACACAAGTCA	
*COQ9*	XM_005899884.2	F: ATACCCGCCGAGCAGT	164
		R: CTCTCCGGTGGACTTGACCT	
*COX4*	XM_005892420.2	F: TGGCAACCAGAGTATTTAGCCT	127
		R: TAGTCACGCCGGTCCAC	
*ATP5F1*	XM_005905667.2	F: TGGTTCAAAAGCGCCAT	160
		R: TGTTCCTTCTGACGCATC	
*LDHA*	XM_014479751.1	F: AATACAGCCCAAATTGCAAG	70
		R: TTCCAAGCCACATAGGTCA	

*COQ9*: Coenzyme Q9; *COX4*: Cytochrome c oxidase subtype IV; *ATP5F1*: ATP synthase F(0) complex subunit B1; *LDHA*: lactate dehydrogenase A; *18S*: 18S rRNA.

**Table 2:** The differentially expressed proteins (DEPs) in ruminal epithelium between GRY and GNY.

No.	Accession Number	Protein ID	Gene ID	GRY/GNY Ratio	*p*-Value
1	XP014338291.1	Collagen alpha-1(VII) chain	COL7A1	2.615	0.000
2	XP014332664.1	Urea transporter 1 isoform X2	SLC14A1	2.590	0.004
3	XP005894969.1	Alpha-endosulfine	ENSA	2.178	0.007
4	XP005896989.1	Charged multivesicular body protein 4b	CHMP4B	2.077	0.009
5	XP005888708.1	4-Trimethylaminobutyraldehyde dehydrogenase	ALDH9A1	5.974	0.013
6	XP005897961.1	Gamma-interferon-inducible lysosomal thiol reductase	IFI30	3.025	0.013
7	XP014336324.1	Septin-8 isoform X6	SEPTIN8	3.395	0.013
8	XP005887104.1	26S proteasome non-ATPase regulatory subunit 1	PSMD1	1.834	0.014
9	XP005899315.1	Transmembrane 9 superfamily member 2	TM9SF2	1.952	0.020
10	XP005905989.1	Heterogeneous nuclear ribonucleoprotein H	HNRNPH1	1.509	0.022
11	XP005887576.1	Splicing factor 3A subunit 1	SF3A1	2.443	0.028
12	XP014338421.1	Serine/arginine-rich splicing factor 6	SRSF6	1.512	0.028
13	XP014337469.1	Protein SCO1 homolog, mitochondrial	LOC102285116	2.121	0.031
14	XP005903851.1	elafin	PI3	4.408	0.036
15	XP005891798.1	ER membrane protein complex subunit 2	EMC2	1.666	0.036
16	XP005898898.1	Low molecular weight phosphotyrosine protein phosphatase isoform X2	ACP1	1.748	0.043
17	XP005887779.1	Threonine--tRNA ligase, cytoplasmic isoform X2	TARS1	1.953	0.043
18	XP005903557.1	Lymphocyte-specific protein 1	LSP1	1.555	0.044
19	XP005896048.1	Histone H2A type 1-like	LOC102280777	1.511	0.046
20	XP005901593.1	Dihydrolipoyllysine-residue acetyltransferase component of pyruvate−dehydrogenase complex, mitochondrial isoform X2	DLAT	1.558	0.046
21	XP005911546.1	V-type proton ATPase subunit D	ATP6V1D	0.357	0.001
22	XP005909437.2	Glutathione peroxidase 1	GPX1	0.125	0.002
23	XP005887951.1	Short palate, lung, and nasal epithelium carcinoma-associated protein 2A	LOC102271013	0.349	0.003
24	XP005902818.2	2-oxoisovalerate dehydrogenase subunit beta, mitochondrial	BCKDHB	0.347	0.003
25	XP005906367.1	V-type proton ATPase catalytic subunit A	ATP6V1A	0.491	0.004
26	XP005898873.1	Alcohol dehydrogenase 6	LOC102284246	0.262	0.008
27	XP005895336.1	Hydroxymethylglutaryl-CoA synthase, mitochondrial	HMGCS2	0.593	0.010
28	XP005910791.1	Microtubule-associated protein 4 isoform X2	MAP4	0.381	0.010
29	XP005891129.1	Catenin beta-1	CTNNB1	0.613	0.011
30	XP005896630.1	Cocaine esterase	CES2	0.435	0.011
31	XP005892792.2	basigin	BSG	0.554	0.012
32	XP005905265.1	Alkaline phosphatase, tissue-nonspecific isozyme	ALPL	0.458	0.013
33	XP014334225.1	Prolactin-inducible protein homolog isoform X3	LOC102271568	0.418	0.014
34	XP005897415.1	UDP-glucose 6-dehydrogenase isoform X1	UGDH	0.645	0.015
35	XP005901484.1	Chloride anion exchanger	SLC26A3	0.325	0.016
36	XP005910259.1	Lactadherin isoform X1	MFGE8	0.560	0.016
37	XP005906507.2	Glutathione S-transferase A1	LOC102274807	0.465	0.022
38	XP005900452.1	Acetyl-CoA acetyltransferase, cytosolic isoform X1	ACAT2	0.538	0.023
39	XP005896890.2	Oxygen-dependent coproporphyrinogen-III oxidase, mitochondrial	CPOX	0.520	0.023
40	XP005906127.1	Mitochondrial 2-oxoglutarate/malate carrier protein isoform X3	SLC25A11	0.471	0.025
41	XP014335386.1	Tenascin	TNC	0.552	0.031
42	XP005899319.1	Propionyl-CoA carboxylase alpha chain, mitochondrial isoform X3	PCCA	0.667	0.034
43	XP014335094.1	Ladinin-1	LAD1	0.660	0.034
44	XP014331756.1	1,2-dihydroxy-3-keto-5-methylthiopentene dioxygenase	ADI1	0.393	0.037
45	XP005901352.1	Ferritin heavy chain isoform X2	FTH1	0.145	0.041
46	XP005894555.1	Eukaryotic translation initiation factor 3 subunit C	LOC102275797	0.657	0.042
47	XP005897085.1	Endoplasmic reticulum resident protein 44	ERP44	0.630	0.045
48	XP005889654.1	SCY1-like protein 2	SCYL2	0.554	0.047
49	XP014337951.1	14-3-3 protein eta	YWHAH	0.658	0.048
50	XP005892436.1	Inositol monophosphatase 2	IMPA2	0.420	0.048
51	XP005901633.1	Sulfhydryl oxidase 1	QSOX1	0.592	0.048
52	XP005890379.1	Acyl-CoA synthetase family member 2, mitochondrial isoform X2	ACSF2	0.591	0.049

GRY: growth-retarded yaks. GNY: growth-normal yaks.

**Table 3:** The KEGG pathway analysis of DEPs in ruminal epithelium between GRY and GNY.

Categories	Subcategories	Pathway	DEPs	*p*-Value
Metabolism	Amino acid metabolism	Valine, leucine and isoleucine degradation	BCKDHB↓, HMGCS2↓, ALDH9A1↑, ACAT2↓, PCCA↓	0.002
Metabolism	Metabolism of cofactors and vitamins	Thiamine metabolism	ALPL↓, ACP1↑	0.006
Metabolism	Lipid metabolism	Synthesis and degradation of ketone bodies	HMGCS2↓, ACAT2↓	0.012
Metabolism	Carbohydrate metabolism	Propanoate metabolism	BCKDHB↓, ACAT2↓, PCCA↓	0.018
Metabolism	Carbohydrate metabolism	Pyruvate metabolism	ALDH9A1↑, ACAT2↓, DLAT↑	0.020
Metabolism	Lipid metabolism	Fatty acid degradation	ADH 6↓, ALDH9A1↑, ACAT2↓	0.022
Metabolism	Metabolism of terpenoids and polyketides	Terpenoid backbone biosynthesis	HMGCS2↓, ACAT2↓	0.024
Metabolism	Carbohydrate metabolism	Ascorbate and aldarate metabolism	ALDH9A1↑, UGDH↓	0.024
Organismal Systems	Excretory system	Collecting duct acid secretion	ATP6V1D↓, ATP6V1A↓	0.024
Organismal Systems	Digestive system	Mineral absorption	SLC26A3↓, FTH1↓	0.034
Metabolism	Global and overview maps	Metabolic pathways	ATP6V1D↓, BCKDHB↓, ATP6V1A↓, ADH 6↓, HMGCS2↓, ALDH9A1↑, ALPL↓, UGDH↓, ACAT2↓, CPOX↓, PCCA↓, ADI1↓, ACP1↑, DLAT↑, IMPA2↓	0.039
Metabolism	Carbohydrate metabolism	Glycolysis/Gluconeogenesis	ADH 6↓, ALDH9A1↑, DLAT↑	0.042
Metabolism	Carbohydrate metabolism	Butanoate metabolism	HMGCS2↓, ACAT2↓	0.045
Human Diseases	Cancers: Specific types	Basal cell carcinoma	CTNNB1↓	0.049

DEPs: Differently expressed proteins. ↓: The protein expression downregulated in the ruminal epithelium of growth-retarded yaks. ↑: The protein expression upregulated in the ruminal epithelium of growth-retarded yaks.

**Table 4:** Mitochondrial DNA copy number and metabolites concentrations of ATP generation in the ruminal epithelium of GRY and GNY.

Items	Groups		SEM	*p*-Value
	GRY	GNY		
Mitochondrial DNA copy number	7.27 ^a^	23.73 ^b^	4.994	0.009
α-Ketoglutarate (μg/g)	1.50 ^a^	1.13 ^b^	0.127	0.005
Succinate (mg/g)	4.43	3.84	0.826	0.490
Lactate (μmol/g)	15.89 ^b^	22.13 ^a^	5.627	0.034
AMP (μg/g)	258.75 ^b^	505.18 ^a^	3.731	0.000
ADP (μg/g)	11.16	11.08	0.240	0.766
ATP (μg/g)	49.27 ^b^	63.82 ^a^	0.788	0.000
AMP/ATP	7.92 ^a^	5.25 ^b^	0.117	0.000

^a,b^ Values within a row with different superscripts differ significantly at *p* < 0.05. GRY: growth retard yaks. GNY: growth-normal yaks.

**Table 5:** Activities of key enzymes in ATP generation in the ruminal epithelium of GRY and GNY.

Items	Groups		SEM	*p*-Value
	GRY	GNY		
CS (U/mg)	0.08 ^b^	0.12 ^a^	0.007	<0.001
ICD (U/mg)	0.96 ^b^	1.36 ^a^	0.800	<0.001
α-KGDHC (U/mg)	0.78 ^b^	1.09 ^a^	0.070	0.020
MRCC I (U/g)	1882.25 ^b^	2308.22 ^a^	84.144	0.004
MRCC II (U/g)	2042.14 ^b^	3487.05 ^a^	283.318	0.003
MRCC III (U/g)	264.74 ^b^	367.52 ^a^	19.116	0.001
MRCC IV (U/g)	189.18 ^b^	249.15 ^a^	12.852	0.011
MRCC V (U/g)	581.73 ^b^	858.10 ^a^	55.301	0.005
LDH-A (U/g)	101.01 ^b^	135.74 ^a^	34.728	0.001

CS = citrate synthase. ICD = isocitrate dehydrogenase. α-KGDHC = α-ketoglutarate dehydrogenase complex. MRCC = Mitochondrial respiratory chain complex. GRY: growth-retarded yaks. GNY: growth-normal yaks. ^a,b^ Values within a row with different superscripts differ significantly at *p* < 0.05.

**Table 6:** mRNA expressions of *COQ9*, *COX4*, *ATP5F1* and *LDHA* in the ruminal epithelium between GRY and GNY.

Items	Groups		SEM	*p*-Value
	GRY	GNY		
*COQ9*	1.23 ^b^	5.24 ^a^	0.784	0.029
*COX4*	0.79 ^b^	3.01 ^a^	0.203	0.016
*ATP5 F1*	0.95	1.59	0.299	0.248
*LDHA*	0.99 ^b^	6.43 ^a^	0.899	0.03

GRY: growth retard yaks. GNY: growth-normal yaks. *COQ9*: Coenzyme Q9; *COX4*: Cytochrome c oxidase subtype IV; *ATP5F1*: ATP synthase F(0) complex subunit B1; *LDHA*: lactate dehydrogenase A. ^a,b^ Values within a row with different superscripts differ significantly at *p* < 0.05.

**Table 7:** Correlation analysis between ADG, rumen epithelium morphological parameters, and ATP generation.

Item	Mitochondrial DNA Copy Number	ATP Concentrations	LA Concentrations
ADG	0.772 **	0.728 **	0.182
Rumen weight	0.631 *	0.957 **	0.181
Papillae height	0.186	0.770 **	0.158
Papillae width	0.046	−0.102	−0.418
Papillae surface area	0.208	0.493	−0.223
Muscular thickness	0.018	0.131	0.291

** represented the *p* < 0.01 and * represented the *p* < 0.05. LA: lactate. ADG: average daily gain.

## Data Availability

None of the data were deposited in an official repository. The data and models supporting the results of this study are available from the authors upon request.

## Supplementary Materials

The following supporting information can be downloaded at: https://www.mdpi.com/article/10.3390/ani14081243/s1, Table S1. Gene ontology (GO) analysis of DEPs in ruminal epithelium between GRY and GNY.
