# Supplementary material for: Proteomics Reveals the Obstruction of Cellular ATP Synthesis in the Ruminal Epithelium of Growth-Retarded Yaks
Source: Animals (Basel). 2024 Apr 22;14(8):1243. doi: 10.3390/ani14081243 (PMC11047487; doi:10.3390/ani14081243)
Supplement: Supplementary file 1 [file animals-14-01243-s001.zip › animals-2891120-supplementary.pdf]

Supplementary Table S1. Gene ontology (GO) analysis of DEPs in ruminal epithelium between GRY and GNY.

Categories	Gene Ontology term	Cluster frequency	*P*-value
cellular component (CC)	proton-transporting V-type ATPase complex	2 out of 40 genes, 5.0%	0.024
	plasma membrane	12 out of 40 genes, 30.0%	0.042
molecular function (MF)	steroid hormone receptor binding	2 out of 42 genes, 4.8%	0.017
	nuclear hormone receptor binding	2 out of 42 genes, 4.8%	0.032
	hormone receptor binding	2 out of 42 genes, 4.8%	0.038
	active transmembrane transporter activity	3 out of 42 genes, 7.1%	0.039
biological process (BP)	transition metal ion transport	3 out of 35 genes, 8.6%	0.003
	cellular transition metal ion homeostasis	3 out of 35 genes, 8.6%	0.007
	transition metal ion homeostasis	3 out of 35 genes, 8.6%	0.011
	mRNA splice site selection	2 out of 35 genes, 5.7%	0.013
	regulation of centrosome cycle	2 out of 35 genes, 5.7%	0.013
	response to nutrient	2 out of 35 genes, 5.7%	0.017
	intracellular steroid hormone receptor signaling pathway	2 out of 35 genes, 5.7%	0.017
	steroid hormone mediated signaling pathway	2 out of 35 genes, 5.7%	0.017
	hormone-mediated signaling pathway	2 out of 35 genes, 5.7%	0.021
	cellular cation homeostasis	4 out of 35 genes, 11.4%	0.025
	glutathione metabolic process	2 out of 35 genes, 5.7%	0.025
	cellular ion homeostasis	4 out of 35 genes, 11.4%	0.028
	iron ion transport	2 out of 35 genes, 5.7%	0.030

	intracellular receptor signaling pathway	2 out of 35 genes, 5.7%	0.030
	homeostatic process	7 out of 35 genes, 20.0%	0.030
	cation homeostasis	4 out of 35 genes, 11.4%	0.034
	regulation of alternative mRNA splicing, via spliceosome	2 out of 35 genes, 5.7%	0.035
	tissue homeostasis	2 out of 35 genes, 5.7%	0.035
	ion transport	5 out of 35 genes, 14.3%	0.038
	inorganic ion homeostasis	4 out of 35 genes, 11.4%	0.038
	regulation of RNA splicing	3 out of 35 genes, 8.6%	0.039
	metal ion transport	3 out of 35 genes, 8.6%	0.042
	cellular chemical homeostasis	4 out of 35 genes, 11.4%	0.042
	cellular homeostasis	5 out of 35 genes, 14.3%	0.043
	ion homeostasis	4 out of 35 genes, 11.4%	0.045
	cellular iron ion homeostasis	2 out of 35 genes, 5.7%	0.046
	cation transport	4 out of 35 genes, 11.4%	0.050
